# Population Genetic Diversity and Species Distribution Evaluation of *Bletilla striata* (Orchidaceae) in Southwest China Using SSR Markers

**DOI:** 10.1002/ece3.72043

**Published:** 2025-08-19

**Authors:** Liangliang Luo, Qian Wang, Xiaolan Li, Delin Xu, Huan Hu

**Affiliations:** ^1^ Microbial Resources and Drug Development Key Laboratory of Guizhou Provincial Department of Education, School of Stomatology Zunyi Medical University Zunyi China; ^2^ School of Preclinical Medicine Zunyi Medical University Zunyi China

**Keywords:** *Bletilla striata*, Maxent model, population genetic structure, potentially suitable habitats, simple sequence repeat (SSR)

## Abstract

We assessed the genetic diversity and population structure of the protected orchid *Bletilla striata* across 18 wild populations in southwestern China. Eight pairs of simple sequence repeat (SSR) molecular markers were employed for its genetic diversity and population structure analyses, while the optimized Maxent model was utilized to predict changes in the habitat distribution under historical conditions and three future climate scenarios (SSP126, SSP245, and SSP585) with 141 natural distribution data and 19 climatic factors. The results revealed an average number of alleles (*Na*) of 3.549 and an effective number of alleles (*Ne*) of 2.636, with a mean polymorphic information content (*PIC*) of 0.748 across the *B. striata* populations. Moderate genetic diversity was observed (observed heterozygosity, *Ho* = 0.402; expected heterozygosity, *He* = 0.509), with 73% of the total variation found within populations, while the 02 population in Zhijin Guizhou exhibited relatively high genetic differentiation (*Ho* = 0.675, *He* = 0.658). UPGMA clustering, population structure analyses, and principal component analysis identified two primary subgroups within *B. striata* . Among the 19 climate variables analyzed, four temperature‐related factors and two precipitation‐related factors were identified as key drivers influencing the geographical distribution of *B. striata* . Future projections for the 2050s and 2070s under varying climate scenarios indicate a northward expansion of suitable habitats for *B. striata* . The proportion of suitable habitat area is expected to increase from 288.3450 × 10^4^ km^2^ under historical conditions (1970–2000) to 351.9792–405.6077 × 10^4^ km^2^ (2050s–2070s). The wild *B. striata* populations in southwestern China and adjacent regions represent valuable germplasm resources with high genetic diversity, offering significant potential for artificial cultivation initiatives. Moreover, predictions of future distribution dynamics provide critical insights to guide the conservation, development, and sustainable utilization of *B. striata* .

## Graphical abstract

These findings provide a scientific basis for conserving and sustainably utilizing *Bletilla striata* germplasm. By predicting future distribution shifts under climate change, this research offers critical insights for dynamic monitoring and conservation strategy development, holding significant theoretical and practical value for biodiversity conservation.

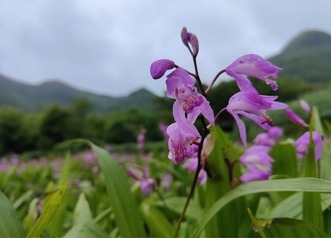

## 1 Introduction

**FIGURE 1:**
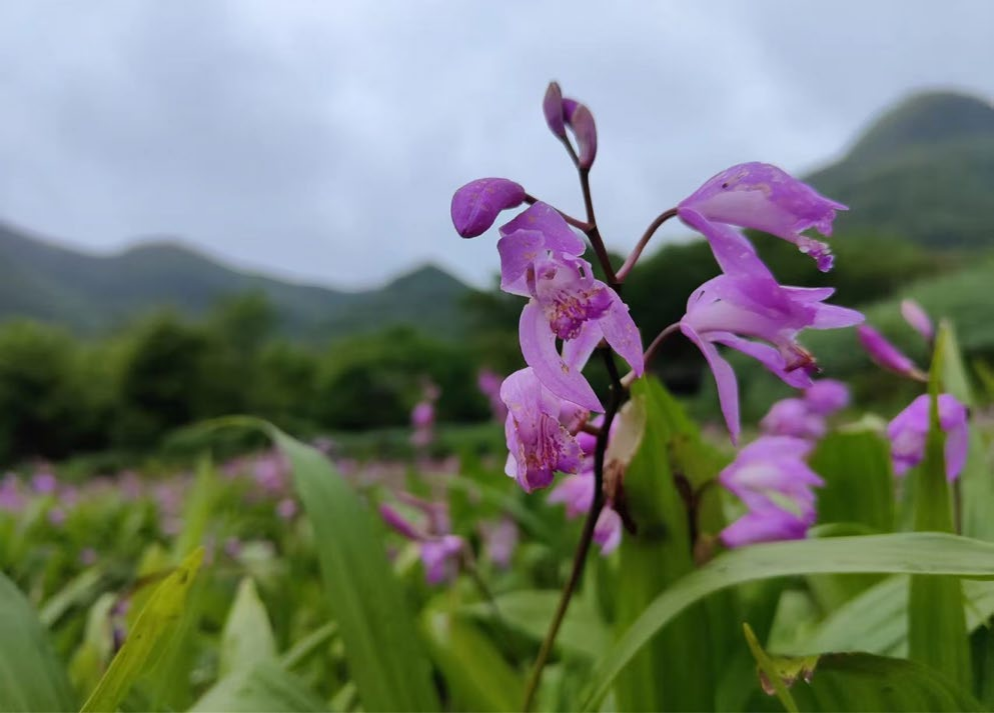
Represent photo of *Bletilla striata* . Photographed by Huan Hu.

The genus *Bletilla* Rchb. f. (Orchidaceae) comprises approximately six species, four of which are native to China, predominantly distributed across southern provinces (Chen et al. 2009). Among these, *Bletilla striata* (Thunb. ex A. Murray) Rchb. F. occupies a prominent role as a traditional medical plant with over 1500 years of documented use in Chinese medicine. It is highly regarded for its pharmacological properties, including hemostatic, anti‐inflammatory, analgesic, and wound‐healing effects (Gou et al. 2022; He et al. 2024). Despite its significance, the propagation of *B. striata* under natural conditions faces considerable challenges due to its tiny seeds, which lack endosperm and hinder direct germination in the wild. This constraint has limited the reproductive success and scale of natural populations. Moreover, the high medicinal value of *B. striata* has driven extensive and destructive harvesting of wild resources, resulting in a growing disparity between market demand and supply (Li et al. 2024) (Figure 1).

Large‐scale cultivation can both meet medicinal demand through standardized production and reduce pressure on wild populations. Population genetic studies provide a valuable approach to assessing the genetic diversity of wild *B. striata* germplasm and evaluating genetic variation among populations. Such research establishes a scientific basis for resource conservation and germplasm identification and facilitates the development of effective strategies for selecting superior germplasm for agricultural production. These efforts will collectively contribute to the sustainable development of the *B. striata* industry.

The application of DNA molecular markers, such as Random Amplified Polymorphic DNA (RAPD), Restriction Fragment Length Polymorphism (RFLP), Amplification Refractory Mutation System (ARMS), Cleaved Amplified Polymorphic Sequence (CAPS), Amplified Fragment Length Polymorphism (AFLP), DNA Amplification Fingerprinting (DAF), Inter‐Simple Sequence Repeat (ISSR), and Simple Sequence Repeat (SSR), offers a cost‐effective and reliable approach for uncovering the mechanisms underlying genetic diversity and evolutionary dynamics of flora and fauna populations. SSR molecular markers, also referred to as microsatellite DNA or SSRs, are based on sequence length polymorphisms (Jin et al. 2024). These markers consist of DNA fragments formed by one or more tandemly repeated nucleotide sequences and, along with single nucleotide polymorphism (SNP) markers, are widely used to analyze population genetic structure and phylogenetic relationships (Tautz and Renz 1984). With their high polymorphism, stability, and codominance, SSR markers have been extensively applied in studies of genetic diversity and population structure in resource plants such as *Passiflora edulis* Sims (Wu, Cai, et al. 2024a), *Habenaria* Willd. (Zhang et al. 2024), *Orchidantha chinensis* T. L. Wu (Zhou et al. 2024), and *Meconopsis integrifolia* (Maxim.) Franch. (Wu, Yang, et al. 2024b). However, research on the genetic structure of wild *B. striata* populations using SSR markers remains largely unexplored. Our previous work has established a foundation by conducting RNA sequencing of *B. striata* tissues and organs at different development stages (Xu et al. 2018). From this, SSR primers were developed and validated, demonstrating their utility for phylogenetic research and breeding programs.

Moreover, climate change driven by increasing greenhouse gas emissions, coupled with the frequent occurrence of extreme weather events, significantly impacts the potential distribution, spatial patterns, and sustainability of global ecosystems (Zhang et al. 2021; Habibullah et al. 2022). The species distribution model (SDM) has become a widely used mathematical approach for predicting species distribution under changing climate conditions (Wan et al. 2024). Among these, the Maxent model, proposed by Jaynes in 1957 based on entropy theory (Phillips et al. 2006), is a machine learning algorithm that offers high flexibility in processing various data types. Its predictive accuracy remains stable and reliable even when data are partially missing or sample sizes are small, making it extensively applied in ecological studies (Remya et al. 2015; Li et al. 2020).

This study integrates SSR markers and SDMs to systematically reveal the genetic structure and climate adaptability of *Bletilla striata* in Southwest China, providing a dual “genetic‐ecological” basis for conservation and sustainable utilization of this endangered orchid. The primary purpose of the present study was to (1) explore the population diversification and structure throughout southwestern China and its adjacent areas, (2) project the potential suitable habitats and centroid migration of *B. striata* under different future climate scenarios, (3) uncover the patterns of suitable habitat redistribution in *B. striata* populations in response to climate changes.

## 2 Materials and Methods

### 2.1 Wild *B. striata* Samples

#### 2.1.1 Plant Material and DNA Extraction

In this study, 116 samples from 18 populations of *B. striata* were gathered from the southwest mountainous region of China and its adjacent areas, which represent the natural distribution center of *B. striata* (Table 1). The field sample collection and research procedures were approved by the Ethical Experimentation Committee of Zunyi Medical University (Identification Code: ZMU‐BO‐2003‐021) and adhered to the legal and ethical criteria set by the local government. The original specimens were identified as *B. striata* by Professor Delin Xu of Zunyi Medical University, and a voucher specimen was deposited in the Herbarium of Life Science Museum at Zunyi Medical University. Fresh young leaves were collected and dried using silica gel. The entire DNA was extracted using the Tiangen Plant Genome DNA Extraction Kit, electrophoresed on a 2% agarose gel, and stored at −20°C for further use.

**TABLE 1:** The sampling sites and genetic diversity information based on eight pairs of simple sequence repeats (SSR) for *Bletilla striata* populations collected in this study.

Sample size (*N*)	Population code	Collection locations	Longitude(E)	Latitude(N)	*Na*	*Ne*	*I*	*Ho*	*He*
8	01	Gouba Village, Zunyi, Guizhou	106.5643	27.6516	3.125	2.209	0.842	0.313	0.479
5	02	Zhongzhai Town, Zijin, Guizhou	105.5964	26.6949	4.000	3.378	1.226	0.675	0.658
8	03	Xifeng Town, Guizhou Province	106.596	27.246	4.000	2.810	1.072	0.438	0.576
5	04	Luokuangyan, Wushan, Chongqing	110.0506	31.1527	3.250	2.676	0.953	0.475	0.543
8	05	E'meishan Biological Station, Sichuan	103.3797	29.5922	4.500	2.850	1.075	0.422	0.535
4	06	Xicao Village, Wenchuan, Sichuan	103.283	30.8899	2.750	2.256	0.733	0.250	0.406
2	07	Qingcheng Mountain, Dujiangyan, Sichuan	103.5548	30.9059	2.250	2.008	0.671	0.438	0.422
4	08	Longzhu Village, Wenchuan, Sichuan	103.3473	30.8985	3.125	2.506	0.926	0.375	0.520
8	09	Huoshiba Village, Zheng'an, Guizhou	107.3578	28.8309	3.500	2.620	0.912	0.172	0.484
8	10	Yilong, Nanchong, Sichuan	106.8067	31.4369	4.125	2.506	0.912	0.266	0.461
8	11	Wawushan Town, Hongya, Sichuan	102.9986	29.6742	3.500	2.400	0.878	0.156	0.459
8	12	Laojun Mountain, Yibin, Sichuan	103.986	28.6916	3.750	3.214	0.965	0.656	0.516
8	13	Houhe Tianmen Gorge, Yichang, Hubei	110.6125	30.0827	5.250	3.395	1.298	0.578	0.639
6	14	Xintang Town, Enshi, Hubei	109.8738	30.1961	3.875	2.775	1.094	0.479	0.590
8	15	Qingliangfeng, Lin'an, Zhejiang	118.959	30.0748	3.750	2.986	0.991	0.359	0.503
8	16	Dazhai Village, Zijin, Guizhou	105.6583	26.4541	4.875	3.327	1.292	0.469	0.653
4	17	Lütang Town, Dafang, Guizhou	105.3978	27.059	1.875	1.652	0.439	0.375	0.273
6	18	Langya Mountain, Chuzhou, Anhui	118.2804	32.2834	2.375	1.874	0.695	0.333	0.438
	Mean				3.549	2.636	0.943	0.402	0.509

*Note: Na*: number of alleles, *Ne*: number of effective alleles, *I*: Shannon diversity index, *Ho*: observed heterozygosity, *He*: expected heterozygosity.

#### 2.1.2 SSR‐PCR Amplification

Based on previous studies, eight pairs of SSR primers were selected and synthesized by Biotech Bioengineering (Shanghai) Co. The PCR reaction system consisted of 1 μL of template DNA, 1 μL each of forward and reverse primers (10 μmol/L), 12.5 μL of 2 × SanTaq PCR Mix (including Mgcl_2_, dNTP, Taq DNA Polymerase, PCR buffer, loading buffer, and PCR enhancer), and 9.5 μL of ddH_2_O. PCR amplification began with 94°C for 3 min, followed by 35 cycles of 94°C for 30 s, annealing at 57°C–58.7°C for 30 s, extension at 72°C for 30 s, and a final extension at 72°C for 7 min (with specific primer information and annealing temperatures provided in Table S1). The amplified products were subsequently sent to Biotech Bioengineering (Shanghai) Co. for capillary electrophoresis gene detection using the 3730XL platform.

#### 2.1.3 Population Genetic Analysis

After analyzing the SSR results using Excel software, GenAlEx 6.503 was employed for AMOVA analysis, principal component analysis (PCoA), and the calculation of genetic diversity parameters. These parameters included the average number of alleles (*Na*), effective alleles (*Ne*), observed heterozygosity (*Ho*), expected heterozygosity (*He*), Shannon's information index (*I*), Nei's genetic identity (*GI*) and distance (*GD*), inbreeding coefficient (*F*is), and genetic differentiation coefficient (*F*st). Gene flow (Nm) was estimated using Wright's formula (Nm = 0.25 (1‐*F*st)/*F*st) (Wright 1931; Rousset 1997; Peakall and Smouse 2012). PowerMarker v3.25 was utilized to calculate polymorphic information content (*PIC*) and conduct UPGMA cluster analysis (Liu and Muse 2005). Additionally, Structure v2.3.4 was used to investigate the population structure of 116 *B. striata* samples from 18 populations (Pritchard et al. 2000) and genetic similarity weight value (Q). The parameters were set as follows: true number of populations (*K*) ranged from 1 to 10, with 20 replicates for each *K*, a length of burn‐in period = 500 000, number of MCMC repeats after burn‐in = 1 500 000. StructureSelector (https://lmme.ac.cn/StructureSelector/) was employed to visualize the optimal segmentation *K* value and the population genetic structure using Evanno's ΔK method (Li and Liu 2018).

### 2.2 Ecological Niche Modeling

#### 2.2.1 *B. striata* Distribution Data and Climate Variables Selecting

**FIGURE 2:**
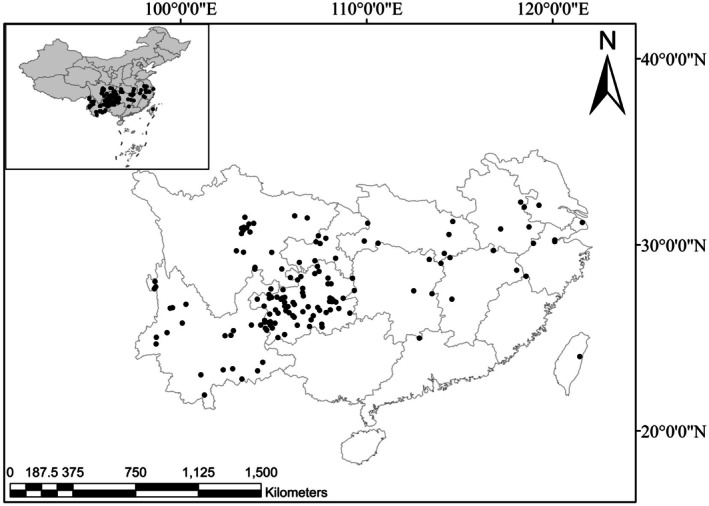
The geographical distribution of 141 recorded occurrence sites of *Bletilla striata* . The occurrence points are presented in black points.

Based on the coordinate information of 18 wild populations collected during field surveys conducted from 2021 to 2022 (Table 1), along with data from the Global Biodiversity Information Facility (GBIF, http://www.gbif.org), the Chinese Virtual Herbarium (CVH, http://www.cvh.org.cn/), and published literature (Deng et al. 2019; Ling et al. 2019; Su et al. 2014; Sun 2015; Sun et al. 2016; Qin et al. 2020; Zhou et al. 2010, 2022; Jian et al. 2019), a preliminary dataset of 185 geographical distribution records of *B. striata* in China was obtained. To reduce bias and prevent overfitting (Bo et al. 2022), the “Trim duplicate occurrences” function in ENM Tools was employed to remove redundant data, resulting in a final dataset of 141 distribution records of *B. straria* (Figure 2).

The map of China was sourced from the National Fundamental Geographic Information System (https://nfgis.nsdi.gov.cn). Climate data for historical (1970–2000), future 2050s (2041–2060), and future 2070s (2061–2080) were obtained from WorldClim (https://worldclim.org/), which includes 19 climate variables. For future scenarios, the BCC‐CSM2‐MR (Beijing Climate Center Climate System Model) climate model from the Coupled Model Intercomparison Project Phase 6 (CMIP6) was selected due to its reliable simulations for China (Liu, Fang, et al. 2023b). Historical climate data were provided at a spatial resolution of 30′, and future climate data for the 2050s and 2070s were modeled at a spatial resolution of 2.5′ under three Shared Socioeconomic Pathways (SSPs): SSP126 (low greenhouse gas emission scenario with radiative forcing of 2.6 W/m^2^ by the year 2100), SSP245 (medium pathway of greenhouse gas emission with 4.5 W/m^2^ in 2100), and SSP585 (high greenhouse gas emission scenario with 8.5 W/m^2^ in 2100).

SPSS Statistics v27 was employed to conduct a bivariate Pearson correlation analysis on 19 climate variables associated with the distribution records. When the correlation coefficient |*r*| ≥ 0.8 between two environmental variables, the one with the higher contribution rate was retained for Maxent model construction (Guo, Cao, Zhang, 2019a) (Figure S1). As a result, six bioclimatic variables (BIO2, BIO4, BIO6, BIO9, BIO12, and BIO17) were selected for further use in Maxent modeling (http://biodiversityinformatics.amnh.org/open_source/maxent/; Table S2).

#### 2.2.2 MaxEnt Model and Suitable Habitat Pattern

Optimizing the feature combination (FC) and regularization multiplier (RM) parameters in the Maxent model can significantly improve the accuracy of predictions (Phillips and Dudík 2008; Radosavljevic and Anderson 2014). The R package Kuenm (Cobos et al. 2019) was employed to explore a total of 248 parameter combinations, consisting of 31 different FC combinations derived from five feature classes (Linear, Quadratic, Product, Threshold, and Hinge), with RM values ranging from 0 to 4 in increments of 0.5. The optimal model was identified when FC = L and RM = 0.5, as this configuration met the model selection criteria, including a data omission rate of less than 5%, statistical significance, and the lowest delta AICc value.

Climate variables for different periods were imported into the Maxent model, with 25% of the distribution data randomly selected for model testing and 75% used for model training. To minimize the effects of random deviations, the bootstrap method with 10 replicates was employed. The “Create response curves” and “Do jackknife” options were activated to assess the relative importance of the climatic variables (Wang et al. 2024). The predictive accuracy of the model was evaluated using the area under curve (AUC) of the receiver operating curve (ROC) (Peterson et al. 2007). An AUC value close to 1 indicates high predictive accuracy, while values exceeding 0.7 are considered to reflect reliable results (Wang et al. 2023).

The predicted distribution map of *B. striata* with the highest AUC value was imported into ArcGIS 10.8 alongside a base map and converted to raster data using ArcToolbox. The reclassify tool was then applied to classify the habitat suitability levels of *B. striata* . Subsequently, the Mask tool in ArcGIS 10.8 was used to extract the potential geographic distribution map of *B. striata* within China. Based on the natural discontinuity point method, the potential suitable areas were categorized into four levels: unsuitable (0–0.12), low‐suitability (0.12–0.25), moderate‐suitability (0.25–0.5), and high‐suitability (0.5–1).

## 3 Results

### 3.1 Genetic Diversity and Structure of *B. striata* in Southwest China and Surrounding Regions

#### 3.1.1 Genetic Diversity

A total of 511 alleles were identified using eight SSR primer pairs across 18 populations of *B. striata* comprising 116 samples from southwest China and surrounding regions. The *Na* varied from 1 to 12, with a mean of 3.549. The *PIC* values ranged from 0.535 to 0.910, with an average of 0.748 (Table S3).

At the population level, genetic diversity indices, including *Na*, *Ne*, *I*, *Ho*, and *He*, ranged from 1.875 to 5.250, 1.652 to 3.395, 0.439 to 1.298, 0.156 to 0.675, and 0.273 to 0.658, respectively. Among the 18 populations, population 11 exhibited the lowest genetic diversity, whereas population 02 displayed the highest genetic diversity (Table 1).

#### 3.1.2 Genetic Differentiation

The *Fst* values among *B. striata* populations ranged from 0.06 (population 06 vs. population 11) to 0.486 (population 06 vs. population 17), with an average of 0.237. The average *Nm* was estimated at 1.058, suggesting moderate genetic exchange between populations while maintaining a relatively high degree of genetic independence (Table S4). Additionally, genetic relationships among populations, evaluated based on *GD* and *GI*, aligned with the results of the *Fst* and Nm analyses. Populations 06 and 11 exhibited the closest genetic relationship, with the lowest *GD* (0.121) and highest *GI* (0.886). Conversely, populations 07 and 18 displayed the highest *GD* (2.393) and the lowest *GI* (0.091), reflecting a higher degree of genetic differentiation. Similarly, populations 06 and 17 exhibited considerable differentiation, with a *GD* of 2.292 and a *GI* of 0.101 (Table S5).

AMOVA results revealed that 27% of the genetic variation was attributable to differences among populations, 23% to variation among individuals within populations, and the majority, 50%, to variation within individuals (Table 2).

**TABLE 2:** Analysis of molecular variance (AMOVA) based on eight pairs of SSR markers for *Bletilla striata* populations.

Source of variation	df	SS	MS	Variance components	Variation percentage
Among Pops	17	237.458	13.968	0.852	27
Among Indiv	98	297.521	3.036	0.723	23
Within Indiv	116	184.5	1.591	1.591	50
Total	231	719.478		3.17	100

Abbreviations: df, degrees of freedom; MS, mean square; SS, sum of squares.

#### 3.1.3 Population Structure Analysis

**FIGURE 3:**
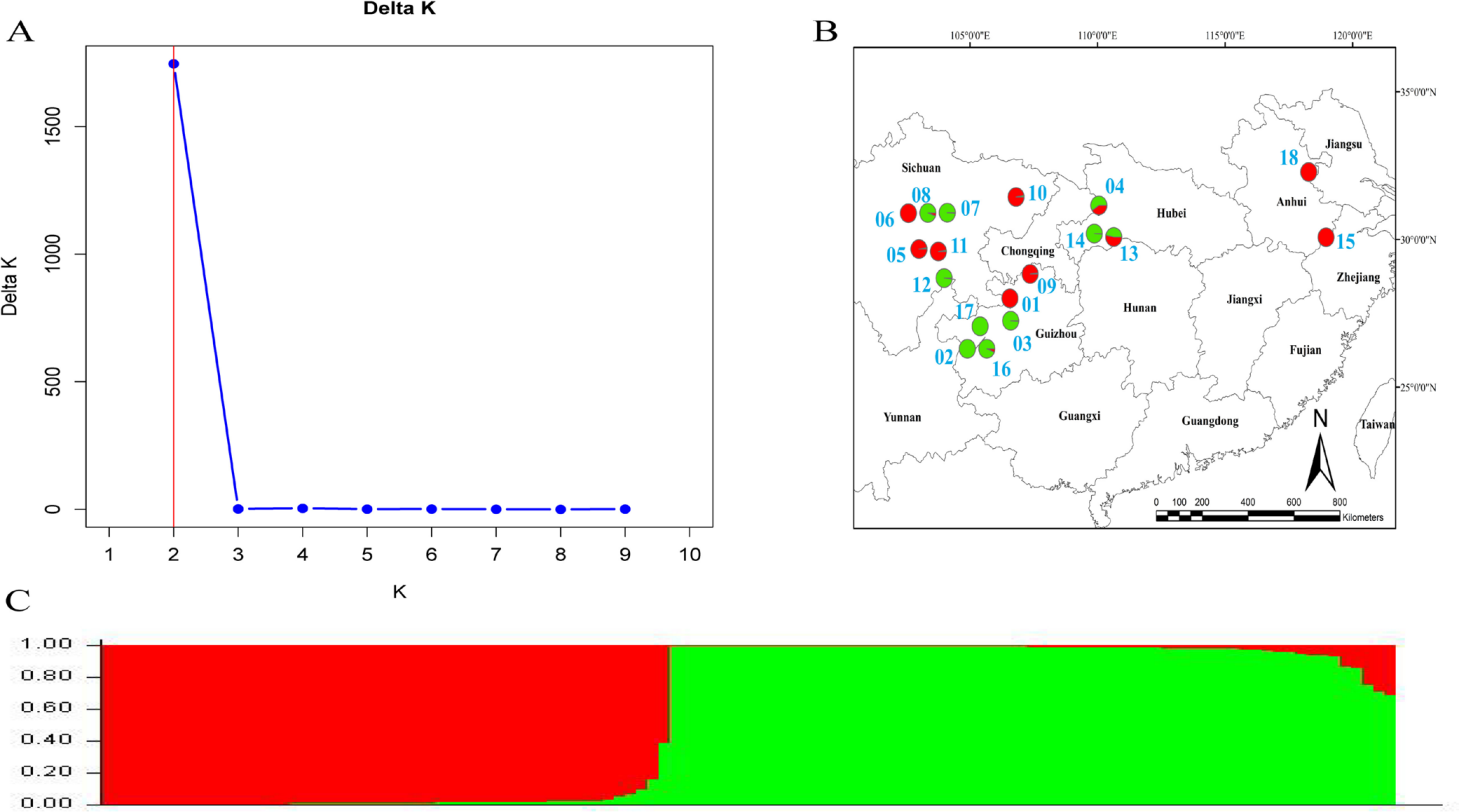
Determination of subpopulations (A) and population genetic structure (B, C) of 116 *Bletilla striata* accessions from 18 populations based on SSR data. (A) Δ*K*. (B) When *K* = 2, the geographical distribution of 18 populations. (C) Genetic clustering result when *K* = 2.

**FIGURE 4:**
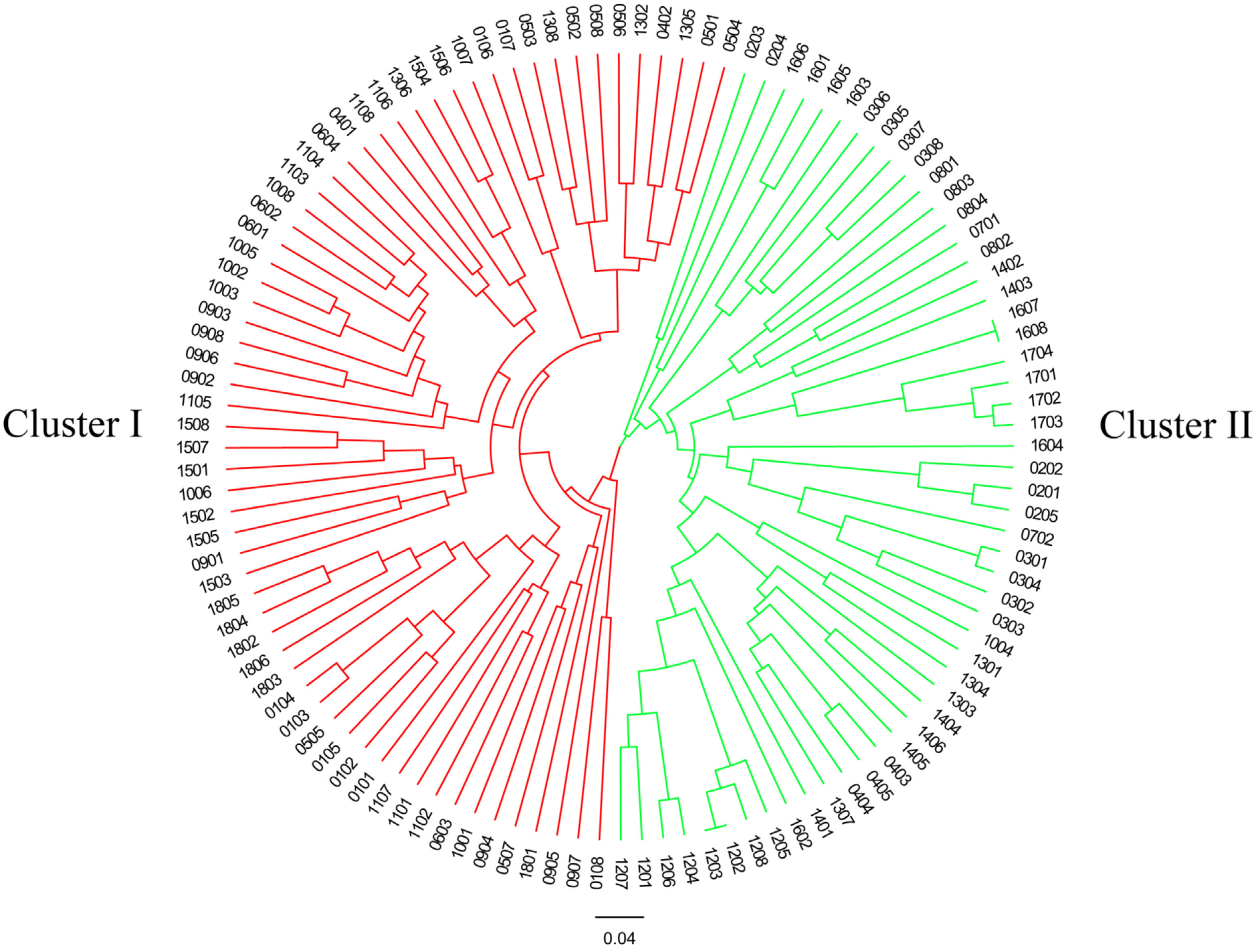
SSR‐based phylogenetic relationships of 116 *Bletilla striata* accessions based on UPGMA cluster analysis. Cluster I: red and Cluster II: green.

**FIGURE 5:**
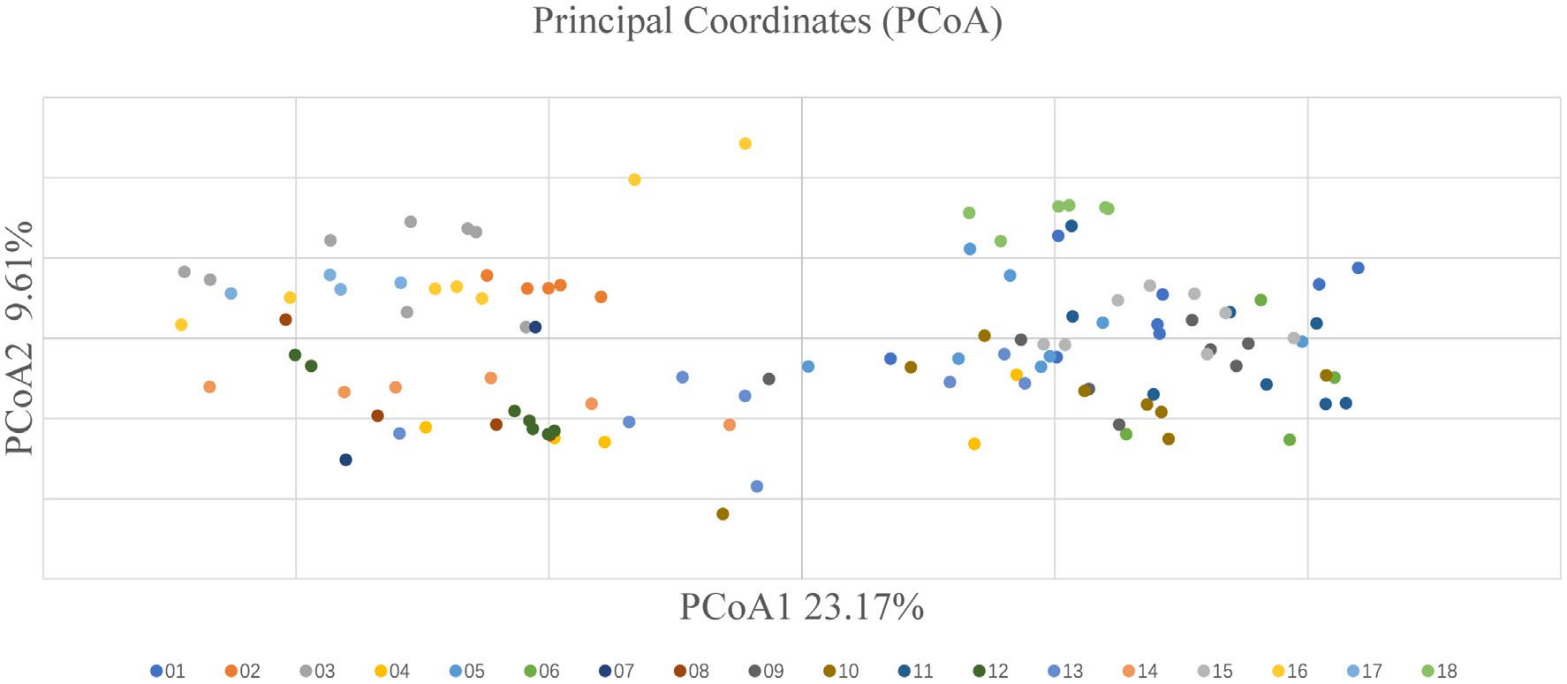
SSR‐based principal coordinate analysis (PCoA) of 116 *Bletilla striata* individuals from 18 populations. Different color points represent *B. striata* populations.

The population genetic structure of 116 *B. striata* samples was analyzed using STRUCTURE analysis, UPGMA clustering, and PCoA, all of which consistently identified two distinct subgroups. StructureSelector identified *K* = 2 as optimal, suggesting two primary genetic clusters: Cluster I (red) and Cluster II (green) (Figure 3). Cluster I consisted of 63 individuals primarily originating from populations 01, 05, 06, 09, 10, 11, 15, and 18, while Cluster II included 53 individuals predominantly from populations 02, 03, 04, 07, 08, 12, 14, 16, and 17. Four individuals had a *Q* value of < 0.8, indicating complex or mixed genetic origins. UPGMA clustering analysis supported the division of the 116 samples into two main subgroups (Figure 4). However, individuals from the same geographic location did not consistently cluster together. For instance, individuals from populations 04, 10, and 13 were distributed across different evolutionary subclades. PCoA analysis showed that PCoA1 contributed 23.17%, and PCoA2 contributed 9.61% of the total variation. The scatter plot exhibited two distinct clusters, with individuals from various populations scattered across the distribution (Figure 5).

### 3.2 Distribution Pattern and Change Prediction

#### 3.2.1 Evaluation of the Maxent Model and Key Bioclimate Variables

The AUC values for various historical and future climate scenarios consistently exceeded 0.9, highlighting the robust predictive accuracy of the optimized Maxent model in reconstructing the population dynamics of *B. striata* . The sole exception was the 2070‐SSP585 climate scenario, which achieved an AUC of 0.898 (Table 3).

**TABLE 3:** AUC mean values of the simulated model of *Bletilla striata* under historical and six future climate scenarios.

	History	2050s			2070s		
		SSP126	SSP245	SSP585	SSP126	SSP245	SSP585
AUC	0.91	0.901	0.919	0.912	0.919	0.915	0.898

Pearson correlation analysis identified six bioclimate variables that significantly contributed to the Maxent model's predictions, with the three most influential being the mean diurnal range (BIO2, 46.6%), min temperature of the coldest month (BIO6, 26.1%), and temperature seasonality (BIO4, 19%). Collectively, these variables accounted for 91.7% of the Maxent model's predictive power (Table S2). Jackknife tests further corroborated these findings, showing that BIO2 had the highest independent impact on the model gain, followed by BIO6 (Figure S2).

To further elucidate the influence of these bioclimatic factors in shaping the potential distribution of *B. striata* , response curves were constructed. These curves demonstrate that when an occurrence probability exceeding 0.5 signifies favorable conditions for plant growth, it thus enables the determination of the optimal bioclimatic range (Yan et al. 2021; Fang et al. 2024).

The response curves for the key climatic variables exhibited the following trends:

- BIO2: Occurrence probability begins to decline when values exceed 4.9°C, stabilizing around 18.267°C.
- BIO6: The curve rises from −8°C, peaking and stabilizing at 16.8°C.
- BIO4: Occurrence probability decreases beyond 306.716°C, reaching equilibrium at 1762.355°C.

Based on these results, the optimal bioclimatic ranges for *B. striata* in its potential habitat are as follows: BIO2 (3.56°C–9.33°C), BIO6 (0.95°C–22.24°C), and BIO4 (161.15°C–679.24°C) (Figure S3).

#### 3.2.2 Distribution of *B. striata* Under Historical Climatic Conditions

**FIGURE 6:**
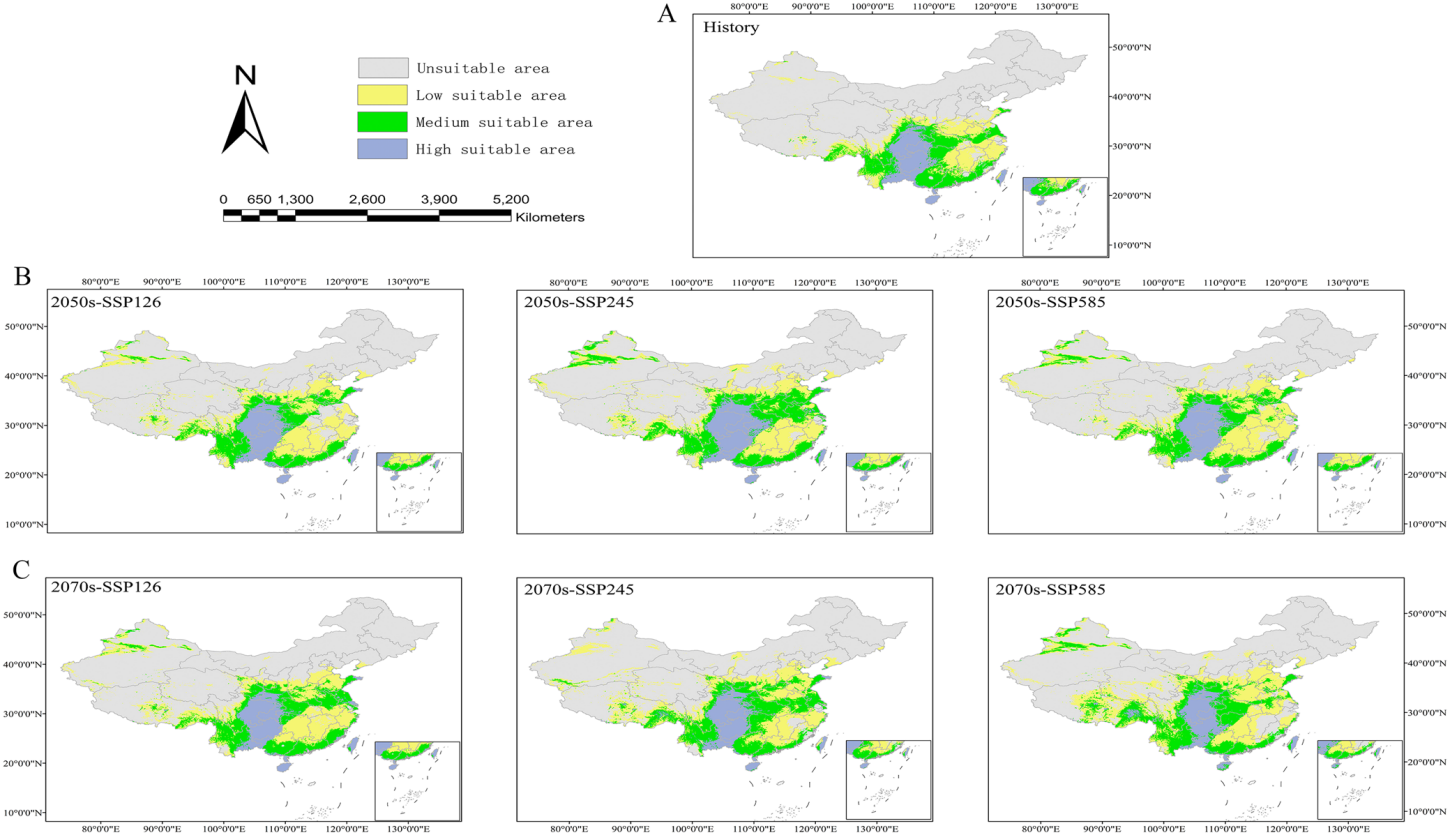
The potential suitable areas for *Bletilla striata* under different climatic scenarios. The prediction was conducted using Maxent modeling in historical (A), 2050s (B), and 2070s (C) periods. Yellow, green, and violet dust areas represent unsuitable, low suitable, medium suitable, and high suitable areas, respectively.

During the 1970–2000 period, the highly suitable habitat for *B. striata* within China was estimated to encompass 67.3099 km^2^, with moderately suitable areas covering 104.7786 km^2^ and low‐suitability areas spanning 116.2565 km^2^. In contrast, unsuitable areas accounted for 670.6716 km^2^. Overall, the total suitable habitat constituted approximately 30.12% of China's land area (Table 4). The highly suitable regions were primarily distributed across southeastern and southwestern China, including Chongqing, Guizhou, western Guangxi, southeastern Yunnan, Hainan, and eastern Taiwan (Figure 6).

**TABLE 4:** Area (×10^4^ km^2^) and proportion (%) of suitable habitats for *Bletilla striata* under historical and future climate scenarios.

Period	Unsuitable area (%)	Total suitable area (%)	Low suitable area (%)	Medium suitable area (%)	High suitable area (%)
History (1970–2000)	670.6716 (69.88)	288.3450 (30.12)	116.2565 (12.12)	104.7786 (10.93)	67.3099 (7.07)
2050s‐SSP126	607.5052 (63.21)	351.9792 (36.69)	170.8455 (17.81)	107.8542 (11.24)	73.2795 (7.64)
2050s‐SSP245	559.8924 (58.35)	399.5921 (41.65)	169.3056 (17.64)	141.7205 (14.77)	88.56560 (9.24)
2050s‐SSP585	591.1632 (61.52)	368.3211 (38.38)	178.6007 (18.61)	117.2378 (12.22)	72.4826 (7.55)
2070s‐SSP126	594.2413 (61.94)	365.2430 (38.06)	167.2691 (17.44)	125.6128 (13.09)	72.3611 (7.53)
2070s‐SSP245	575.8993 (59.98)	383.5851 (40.01)	173.2743 (18.06)	139.6615 (14.56)	70.6493 (14.56)
2070s‐SSP585	553.8768 (57.62)	405.6077 (42.25)	201.2240 (20.97)	141.0799 (14.69)	63.3038 (6.59)

#### 3.2.3 Potential Distribution of *B. striata* Under Future Climatic Scenarios

Under future climate scenarios, the potentially suitable growth areas for *B. striata* are projected to expand as fossil fuel consumption increases. Compared to historical climate conditions, the green development pathway (SSP126) predicts 6.57% and 7.94% increases in suitable habitat area by the 2050s and 2070s, respectively. Similarly, under the SSP245 scenario, the potentially suitable area is anticipated to expand by 11.53% and 9.89%, while the SSP585 scenario forecasts increases of 8.26% and 12.13% for the respective periods (Table 4).

In the 2050s, under the SSP126 scenario, the expansion of highly suitable areas is mainly attributed to the transition of low‐suitability regions in northwestern Taiwan and moderate‐suitability regions in eastern Shandong into high‐suitability zones. This indicates a notable northward shift in habitat suitability. Under the SSP245 scenario, highly suitable areas extend into Shaanxi and Hubei provinces, while regions in Shandong, Henan, and Anhui transition from low to moderate suitability. Additionally, suitable areas begin to emerge in northwestern Xinjiang, with a marked increase in moderately suitable regions, reinforcing the northward expansion trend. The SSP585 scenario predicts significant growth in suitability around the Tianshan Mountains in Xinjiang (Figure 6).

By the 2070s, under the SSP126 and SSP245 scenarios, highly suitable areas are projected to increase by 0.46% and 0.32%, respectively. However, under the SSP585 scenario, a slight decrease of 0.48% is observed. Despite these fluctuations, the primary distribution areas remain concentrated in the southwest, with trends consistent with those observed in the 2050s. These include a continued northward expansion and substantial increases in suitable habitats within Xinjiang (Figure 6).

#### 3.2.4 Distribution Changes Between Historical and Future Climate Scenarios

**FIGURE 7:**
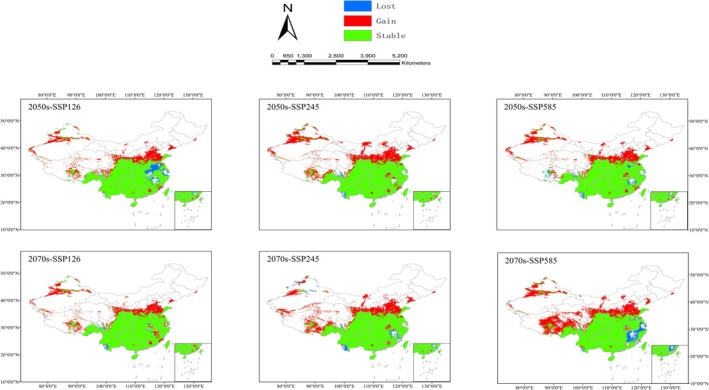
Potential spatial changes of *Bletilla striata* under future climatic scenarios. Blue, red, and green areas represent lost, gain, and stable areas, respectively.

The spatial patterns of suitable habitats for *B. striata* were analyzed by comparing historical distributions with projections for the 2050s and 2070s under different climate scenarios (SSP126, SSP245, and SSP585) (Table 5, Figure 7).

**TABLE 5:** Changes in the suitable habitats of *Bletilla striata* under different future climate scenarios.

Period	Area (×10^4^ km^2^)			Change (%)		
	Gain	Lost	Stable	Gain rate	Loss rate	Stable rate
2050s‐SSP126	81.679	19.387	268.326	22.11	5.25	72.64
2050s‐SSP245	114.901	5.066	282.648	28.54	1.26	70.2
2050s‐SSP585	89.152	10.493	277.217	23.66	2.78	73.56
2070s‐SSP126	80.831	5.179	282.535	21.93	1.41	76.66
2070s‐SSP245	104.734	10.772	276.943	26.69	2.74	70.57
2070s‐SSP585	135.611	19.308	268.412	32.03	4.56	63.40

Under the SSP126 scenario, the growth rates of suitable habitats are projected to be 22.11% and 21.93% for the 2050s and 2070s, respectively. The primary regions contributing to habitat expansion include Hebei, Shandong, Tibet, Shanxi, Shaanxi, and Xinjiang. Habitat loss rates are estimated at 5.25% and 1.41% for the respective periods. In the 2050s, habitat loss is concentrated in northwestern Anhui, eastern Hubei, and southern Henan, while in the 2070s, the losses are mainly observed in southwestern Yunnan.

Under the SSP245 scenario, suitable habitat growth rates are expected to reach 28.54% and 26.69% for the 2050s and 2070s, respectively, with growth occurring in regions similar to those under the SSP126 scenario. However, a rapid expansion trend is projected in Inner Mongolia. Habitat loss rates are 1.26% and 2.74% for the 2050s and 2070s, with losses concentrated in southwestern Yunnan during both periods.

Under the SSP585 scenario, the habitat growth rates are projected at 23.66% for the 2050s and 32.03% for the 2070s. The primary regions driving habitat expansion in the 2050s are similar to those under SSP126 and SSP245, while the 2070s are expected to see significant habitat growth across Tibet. Habitat loss rates are estimated at 2.78% and 4.56% for the 2050s and 2070s, respectively. In the 2050s, habitat loss is concentrated in northeastern Anhui, southwestern Yunnan, and parts of eastern Jiangxi. By the 2070s, losses are expected in southeastern Jiangxi, northwestern Fujian, and northwestern Zhejiang.

Across future climate scenarios, the stability rate for suitable habitats is projected to range from 63.40% to 76.66%. These stable regions are predominantly located in southwestern and central China, indicating areas of consistent suitability under changing climatic conditions.

**FIGURE 8:**
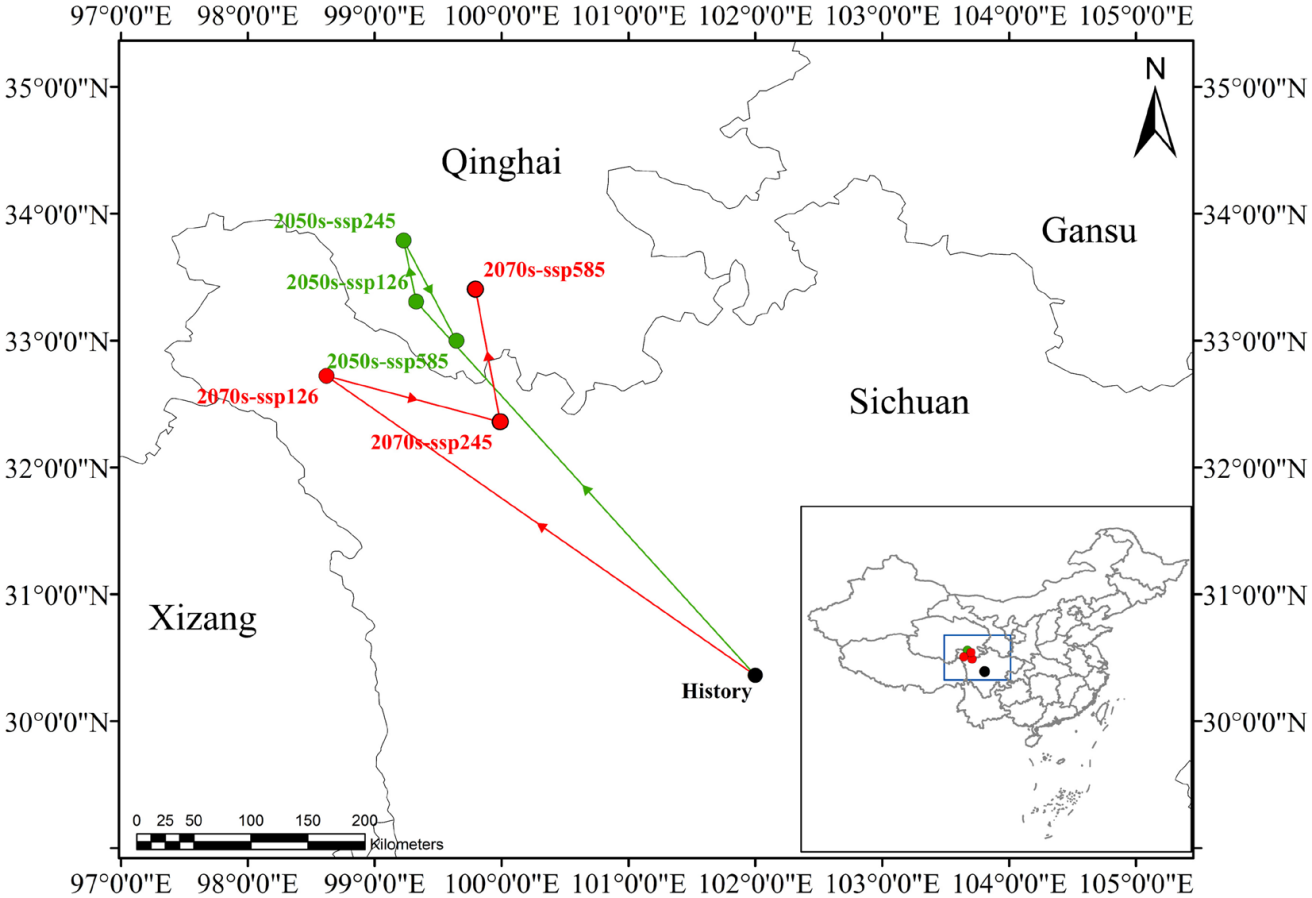
Migration of the center of suitable habitats for *Bletilla striata* migratory routes in historical and future climate scenarios. Black, green, and red dots represent the suitable habitat distribution centroid in historical, 2050s, and 2070s periods, respectively.

Under historical climate conditions, the distribution center of *B. striata* was primarily located in Sichuan Province (101°59′ E, 30°21′ N). In the 2050s, projections under various climate scenarios indicate a northwestern shift to Qinghai Province, with the distribution centers predicted at (99°19′ E, 33°18′ N) for SSP126, (99°13′ E, 33°47′ N) for SSP245, and (99°47′ E, 33°24′ N) for SSP585. By the 2070s, the predicted distribution center under the SSP126 scenario shifts to northwestern Sichuan (98°37′ E, 32°43′ N), while under SSP245, it remains in northwestern Sichuan (99°59′ E, 32°21′ N). For SSP585, the center moves further into southern Qinghai (99°47′ E, 33°24′ N). Overall, these projections suggest a consistent trend of northwestward movement in the distribution center of *B. striata* under future climate scenarios (Figure 8).

## 4 Discussion

SSR molecular markers are widely utilized in genetic breeding, population genetics, and fingerprinting studies in medicinal plants, such as *Zanthoxylum nitidum* (Roxb.) DC. (Zhu et al. 2023), *Paris polyphylla* Sm. (Oliya et al. 2023) and *Polygonatum cyrtonema* Hua (Liu, Cheng, et al. 2023a). Although the genome (Jiang et al. 2022), transcriptome (Xu et al. 2018; Niu et al. 2020), and chloroplast genome (Cai et al. 2020) of *B. striata* have been published, no systematic population genetic studies have been conducted due to challenges in wild sampling. Our research group utilized transcriptome data to identify 25,935 high‐quality SSR primer pairs (Xu et al. 2018) in earlier work.

In this study, 116 individuals from 18 wild populations of *B. striata* across southwestern China and neighboring regions were extensively sampled. Using eight selected SSR primers, the study documented substantial genetic diversity (mean *Na* = 3.549, *Ne* = 2.636, and *I* = 0.943). The pairwise *Fst* and *Nm* values—key parameters for assessing genetic partitioning (Yang et al. 2022)—indicate moderate genetic differentiation among the 18 *B. striata* populations (mean *Fst* = 0.237; Wright 1922) alongside moderate gene flow (mean *Nm* = 1.058; Slatkin 1987). This pattern reflects a dynamic equilibrium between isolation‐by‐distance effects and episodic long‐distance dispersal events, potentially mediated by pollinator behavior or anthropogenic seed transport. The mountainous terrain functions as a “semi‐permeable landscape filter”, where ridges promote drift‐induced differentiation while valley corridors facilitate constrained gene flow, collectively amplifying intra‐population genetic variation (Hesselbarth et al. 2025). These findings are consistent with AMOVA results, which showed that most genetic variation was attributable to within‐population differences. Furthermore, due to the severe depletion of *B. striata* resources in the wild and the associated challenges in sampling, earlier studies employed molecular markers such as Sequence‐Related Amplified Polymorphism (SRAP), Start Codon Targeted Polymorphism (SCoT), and Inter‐Retrotransposon Amplified Polymorphism (IRAP) to explore the genetic diversity of *B. striata* germplasm across multiple regions, albeit with limited sample sizes (Gui et al. 2024; Guo et al. 2018; Sun et al. 2016). These studies consistently demonstrated high genetic diversity and predominantly within‐population genetic variation, aligning with the results of this study.

Additionally, the UPGMA clustering, STRUCTURE analysis, and PCoA analysis conducted in this study were effective in identifying homogeneous clusters among individuals (Elumalai and Srinivasan 2023). The results consistently indicated that the *B. striata* samples from the 18 populations could be broadly classified into two subgroups. Individuals from the same population exhibited close genetic relationships and tended to cluster together. However, individuals from populations in Wushan (04), Yilong (10), and Houhe Tianmen Gorge (13) were dispersed across different subgroups. This pattern may be attributed to several factors, including intra‐population variation, mixed ancestry, historical gene flow, anthropogenic translocation, incomplete lineage sorting due to recent divergence, or the limitations of the markers and analytical methods used (Wu, Yang, et al. 2024b). The absence of strict geographical segregation between clusters suggests extensive historical gene flow events across southwestern China, possibly facilitated by epizoochory or anthropogenic movement of tubers prior to recent habitat fragmentation. The dispersed distribution of populations 04, 10, and 13 across subgroups may also reflect their adaptive potential shaped by hybridization. Hybrid populations may integrate adaptive alleles from distinct lineages through dominance or overdominance effects (Harrison and Larson 2014), thereby increasing genetic diversity and potentially generating more adaptive phenotypes in specific environments. This is particularly relevant for populations in ecotonal zones (e.g., Wushan and Houhe Tianmen Gorge), where fluctuating climates and complex topographies select for phenotypic plasticity (Hoffmann and Sgrò 2011).

Field sampling poses challenges, and our sampling is limited to mainland China, excluding peripheral populations in Korea, Japan, Taiwan, and Hengduan Mountains. While this may affect inferences about species‐wide diversity patterns, prior studies suggest that peripheral populations often exhibit lower genetic diversity due to founder effects and isolation (Tian et al. 2018). The core distribution area (southwestern China) likely retains ancestral diversity, as observed in *B. striata* 's climatic optimal habitats (Gong et al. 2014) and other orchids with glacial refugia in western China (Guo, Cao, Li, et al. 2019b). Future collaborative efforts should prioritize cross‐border sampling to test biogeographic hypotheses. While our SSR markers exhibited high discriminatory power (mean *PIC =* 0.748; Botstein et al. 1980) and association with functional loci (Xu et al. 2018), we acknowledge methodological limitations. The rarefaction curve (Figure S4) indicates incomplete allele saturation, suggesting potential undetected variation with additional markers. Although this constrained marker set aligns with successful orchid diversity studies using 6–10 SSR loci in orchids (e.g., *Zygopetalum mackayi*, Moura et al. 2020; *Vanilla odorata*, Serna‐Sánchez et al. 2025), it may obscure fine‐scale population differentiation. Crucially, the convergence of STRUCTURE, UPGMA, and PCoA results in identifying two primary clusters provides robust validation of our macro‐scale genetic structure conclusions. Future investigations should incorporate more SSR markers or genome‐wide approaches to resolve subtle differentiation patterns and adaptive variation.

Importantly, the observed genetic structure and gene flow patterns may be further influenced by future climate‐driven habitat shifts. Climate change is a key driver of species extinction, habitat redistribution, and shifts in vegetation type, with the Orchidaceae family—one of the most species‐rich plant families worldwide—being particularly vulnerable to its effects (Pica et al. 2024). Our study identified temperature‐related factors as the primary determinants shaping the future distribution patterns of *B. striata* . Specifically, BIO2 (mean diurnal range; 46.6% contribution), BIO6 (min temperature of coldest month; 26.1%) and BIO4 (temperature seasonality; 19%) contributed the most to the Maxent model, with respective contributions of 46.6%, 26.1%, and 19%. The underlying mechanisms may involve the diurnal temperature range affecting biomass accumulation (Sunoj et al. 2016), the minimum temperature during the coldest month facilitating vernalization (Griebeler and Kadereit 2023), and temperature seasonality regulating regular phenological cycles (Zhao et al. 2023). Optimal climatic conditions for *B. striata* growth were found to be BIO2 ranging from 3.56°C to 9.33°C, BIO6 from 0.95°C to 22.24°C, and BIO4 from 161.15°C to 679.24°C. While climate variables were prioritized due to their established strong influence on orchid distributions (Ye et al. 2022; Mafakheri et al. 2022), we acknowledge that excluding factors such as soil properties and topography represents a limitation for habitat suitability generalization (Urban et al. 2016).

This study, based on the CMIP6 model, provides valuable insights into the potential distribution patterns of *B. striata* under future climate scenarios (2050s and 2070s) across SSP126 (low greenhouse gas emissions), SSP245 (moderate emissions), and SSP585 (high emissions). The results indicate a significant expansion of suitable habitats, with coverage increasing from 30.12% under historical conditions to a range of 36.69% (2050s‐SSP126)–42.25% (2070s‐SSP585). The primary suitable habitats of *B. striata* are concentrated in southwestern China, including Guizhou, Chongqing, Sichuan, as well as parts of Hainan, western Guangxi, and southeastern Yunnan. Additionally, its potential distribution extends to areas south of the Qinling Mountains and Huaihe River, with a discernible trend of northward expansion under future climate scenarios. These findings align with those of previous studies (Gong et al. 2014; Wang et al. 2011). However, the actual migration may lag behind climatic suitability due to dispersal limitations and soil‐microbiome dependencies, emphasizing the need for assisted migration strategies (Phillips et al. 2020).

Under projected future scenarios, ecological changes in regions such as Inner Mongolia, Xinjiang, and Tibet may create favorable conditions for the growth of *B. striata* . The distribution center of the species is also expected to shift northwestward, transitioning from Sichuan (1970–2000) to Qinghai (2050s). By the 2070s, the distribution center migrates from central Sichuan to its northwestern regions under SSP126 and SSP245 scenarios and further into Qinghai under SSP585. The northward range expansion may favor Cluster II populations (predominantly northern Sichuan/Yunnan), which exhibit higher allele richness (Na = 11.625 vs. 11.125 in Cluster I) and could harbor pre‐adaptations to cooler environments; experimental studies are needed to test this hypothesis. These projections suggest that northwestern China may become a viable area for the climate‐resilient cultivation of *B. striata* in the future.

These results highlight two key considerations. First, the high genetic diversity observed within natural populations offers critical resources for the conservation of wild *B. striata* . To mitigate human disturbances, we recommend implementing ex situ conservation and reintroduction measures in suitable habitats, particularly in Sichuan and Guizhou. Large‐scale cultivation of *B. striata* in these highly suitable areas offers significant potential for balancing ecological conservation, rational resource utilization, and economic value enhancement. Second, we strongly advocate for establishing long‐term monitoring networks to track population dynamics across the current and projected range, integrating genetic health assessments (e.g., every 5 years) and a threat detection system to enable adaptive management.

## 5 Conclusion

This study integrates SSR‐based population genetics and Maxent species distribution modeling to characterize the genetic architecture and habitat dynamics of *B. striata* across southwestern China and adjacent regions. Key findings reveal 1. a distinct phylogeographic structure with two genetic clusters exhibiting moderate differentiation (*Fst* = 0.237) yet sustained gene flow (*Nm* = 1.058), maintained through a balance of isolation‐by‐distance and episodic long‐distance dispersal across mountainous terrain; 2. significant within‐population diversity (> 80% AMOVA variation), with populations 05 (Cluster I) and 16 (Cluster II) identified as priority genetic reservoirs due to exceptional allelic richness; and 3. climate‐driven distribution shifts dominated by temperature variables (BIO2/4/6), projecting 22%–40% habitat expansion and northwestward centroid migration under future scenarios.

We propose a three‐tiered conservation framework: (1) High‐diversity sources (Pop 05‐Cluster I, Pop 16‐Cluster II) warrant urgent seed banking and serve as primary germplasm for ex‐situ collections, with monitoring for inbreeding depression. (2) Stable founder populations (Pop 01‐Cluster I, Pop 03‐Cluster II) are ideal for cultivation trials in replicable habitats, allowing controlled intraspecific mixing (< 30% admixed propagules) to enhance diversity while preserving local adaptation. (3) Admixed populations (e.g., Pop 04) require spatial isolation in ex‐situ conservation and fitness‐based breeding programs to mitigate outbreeding depression risks.

This work establishes an evolutionarily informed framework for conserving *B. striata* resources under climate change, demonstrating how landscape‐genetic patterns can guide sustainable management of threatened medicinal orchids.

## Author Contributions

**Liangliang Luo:** data curation (equal), software (equal), visualization (equal), writing – original draft (lead). **Qian Wang:** investigation (equal), software (equal), supervision (equal), visualization (equal), writing – review and editing (supporting). **Xiaolan Li:** data curation (equal), formal analysis (equal), resources (equal), software (equal). **Delin Xu:** methodology (equal), project administration (equal), resources (equal), software (equal). **Huan Hu:** funding acquisition (lead), methodology (equal), project administration (equal), supervision (equal), writing – review and editing (lead).

## Conflicts of Interest

The authors declare no conflicts of interest.

## Supporting information


**Appendix S1:** ece372043‐sup‐0001‐AppendixS1.docx.

**Data S1:** ece372043‐sup‐0002‐DataS1.xlsx.

**Data S2:** ece372043‐sup‐0003‐DataS2.xlsx.

## Data Availability

The data and materials supporting this study are included in the article and Appendix S1, Data S1, and Data S2.
