# Supplementary material for: Population Genetic Diversity and Species Distribution Evaluation of Bletilla striata (Orchidaceae) in Southwest China Using SSR Markers
Source: Ecol Evol. 2025 Aug 19;15(8):e72043. doi: 10.1002/ece3.72043 (PMC12362183; doi:10.1002/ece3.72043)
Supplement: Supplementary file 1 — Appendix S1: ece372043‐sup‐0001‐AppendixS1.docx. [file ECE3-15-e72043-s003.docx]

Population genetic diversity and species distribution evaluation of *Bletilla striata* (Orchidaceae) in Southwest China using SSR markers

Liangliang Luo^1,2^, Qian Wang^1^, Xiaolan Li^1^, Delin Xu^2^, Huan Hu^1^

^1^Microbial Resources and Drug Development Key Laboratory of Guizhou Provincial Department of Education, Zunyi Medical University, Zunyi, China

^2^ School of Preclinical Medicine, Zunyi Medical University, Zunyi, China

Running title: Genetic diversity and distribution of *Bletilla striata*

Corresponding Author:

Microbial Resources and Drug Development Key Laboratory of Guizhou Provincial Department of Education, Zunyi Medical University, No.6 Xuefu West Road, Xinpu New District, Zunyi 563000, China.

**E-mail addresses:** Qian Wang, [qianwang07@126.com](mailto:qianwang07@126.com); Huan Hu, [huhuan1990@163.com](mailto:huhuan1990@163.com).

Table S1. Information for eight pairs of polymorphic SSR primers screened.

| Primer | Annealing temperature | Sequence information (5’--3’） | Repeat motifs |
| --- | --- | --- | --- |
| BS-ssr1 | 57℃ | TTGTCACTGGTCGTTGCAGT | (T)15 |
| BS-ssr2 | 58℃ | GCTGCTTTCCATTTCCAGCC | (T)13 |
| BS-ssr15 | 58.7℃ | CCCCCTTTCATCGGATTCCC | (CT)7 |
| BS-ssr20 | 58℃ | CCACCGCAAATCCAGAGAGA | (TG)9 |
| BS-ssr29 | 57℃ | AGCAAAGGACGCTTCAGTCT | (TC)6 |
| BS-ssr51 | 58℃ | AGGAGGACGCTGGAGAGAAT | (ATG)6 |
| BS-ssr62 | 57℃ | GGTGCTTGTTGGTTTCGGTT | (GAA)6 |
| BS-ssr86 | 58℃ | CTCTCGGCCGATGATCTGAG | (ATAA)5 |

Table S2. Climate variables used for Maxent modeling and their percentage contribution.

| Variable | Description | Contribution(%) | Units |
| --- | --- | --- | --- |
| BIO2 | Mean Diurnal Range (Mean of monthly (max temp - min temp)) | 46.6 | °C |
| BIO6 | Min Temperature of Coldest Month | 26.1 | °C |
| BIO4 | Temperature Seasonality (standard deviation ×100) | 19 | °C |
| BIO9 | Mean Temperature of Driest Quarter | 5.3 | °C |
| BIO12 | Annual Precipitation | 1.8 | mm |
| BIO17 | Precipitation of Driest Quarter | 1.3 | mm |

Table S3. Detection information of eight pairs of polymorphic SSR primers.

| Marker | *Na* | *Ne* | *Ho* | *He* | *I* | *F*is | *F*st | *PIC* |
| --- | --- | --- | --- | --- | --- | --- | --- | --- |
| BS-ssr1 | 3.556 | 2.668 | 0.444 | 0.540 | 0.982 | 0.179 | 0.365 | 0.832 |
| BS-ssr2 | 5.389 | 3.649 | 0.637 | 0.699 | 1.428 | 0.090 | 0.214 | 0.876 |
| BS-ssr15 | 2.222 | 1.740 | 0.343 | 0.356 | 0.578 | 0.037 | 0.422 | 0.535 |
| BS-ssr20 | 2.722 | 2.138 | 0.583 | 0.486 | 0.803 | -0.199 | 0.312 | 0.651 |
| BS-ssr29 | 2.722 | 2.053 | 0.323 | 0.363 | 0.661 | 0.112 | 0.467 | 0.614 |
| BS-ssr51 | 4.611 | 3.511 | 0.424 | 0.591 | 1.186 | 0.284 | 0.347 | 0.905 |
| BS-ssr62 | 3.222 | 2.203 | 0.328 | 0.451 | 0.815 | 0.272 | 0.373 | 0.664 |
| BS-ssr86 | 3.944 | 3.125 | 0.132 | 0.581 | 1.091 | 0.773 | 0.367 | 0.910 |
| Mean | 3.549 | 2.636 | 0.402 | 0.509 | 0.943 | 0.193 | 0.358 | 0.748 |

Notes: *Na*: number of alleles, *Ne*: number of effective alleles, *Ho*: observed heterozygosity, *He*: expected heterozygosity, *I*: Shannon diversity index, *Fis*: inbreeding coefficients, *Fst*: genetic differentiation coefficient, *PIC*: polymorphic information content.

Table S4. Gene flow (above) and genetic differentiation coefficient (below) among *B. striata* populations.

| Population | 01 | 02 | 03 | 04 | 05 | 06 | 07 | 08 | 09 | 10 | 11 | 12 | 13 | 14 | 15 | 16 | 17 | 18 |
| --- | --- | --- | --- | --- | --- | --- | --- | --- | --- | --- | --- | --- | --- | --- | --- | --- | --- | --- |
| 01 |  | 0.775 | 0.719 | 0.879 | 2.356 | 1.153 | 0.490 | 0.547 | 1.569 | 1.555 | 1.410 | 0.646 | 1.246 | 0.715 | 2.047 | 0.827 | 0.342 | 1.668 |
| 02 | 0.244 |  | 1.712 | 0.985 | 0.932 | 0.630 | 0.766 | 1.161 | 0.910 | 0.815 | 0.723 | 0.892 | 1.356 | 1.334 | 0.880 | 1.959 | 0.607 | 0.874 |
| 03 | 0.258 | 0.127 |  | 0.993 | 0.850 | 0.515 | 0.799 | 1.031 | 0.795 | 0.725 | 0.598 | 1.034 | 1.233 | 1.628 | 0.717 | 2.476 | 0.756 | 0.809 |
| 04 | 0.221 | 0.202 | 0.201 |  | 1.044 | 0.902 | 0.629 | 0.968 | 1.171 | 1.226 | 0.947 | 1.348 | 3.875 | 3.072 | 0.966 | 1.135 | 0.587 | 0.721 |
| 05 | 0.096 | 0.212 | 0.227 | 0.193 |  | 1.057 | 0.521 | 0.634 | 1.776 | 1.687 | 1.205 | 0.788 | 2.260 | 0.813 | 1.953 | 1.027 | 0.386 | 1.346 |
| 06 | 0.178 | 0.284 | 0.327 | 0.217 | 0.191 |  | 0.368 | 0.470 | 1.672 | 1.881 | 3.888 | 0.489 | 1.199 | 0.658 | 1.395 | 0.612 | 0.264 | 0.792 |
| 07 | 0.338 | 0.246 | 0.238 | 0.284 | 0.324 | 0.405 |  | 1.500 | 0.683 | 0.537 | 0.425 | 0.945 | 0.800 | 0.973 | 0.511 | 0.806 | 0.341 | 0.403 |
| 08 | 0.313 | 0.177 | 0.195 | 0.205 | 0.283 | 0.347 | 0.143 |  | 0.643 | 0.732 | 0.508 | 0.985 | 1.249 | 1.503 | 0.487 | 1.191 | 0.512 | 0.517 |
| 09 | 0.137 | 0.216 | 0.239 | 0.176 | 0.123 | 0.130 | 0.268 | 0.280 |  | 3.202 | 1.784 | 0.665 | 1.677 | 0.873 | 2.683 | 0.880 | 0.341 | 1.193 |
| 10 | 0.138 | 0.235 | 0.256 | 0.169 | 0.129 | 0.117 | 0.318 | 0.255 | 0.072 |  | 1.537 | 0.639 | 1.852 | 0.923 | 1.688 | 0.862 | 0.385 | 0.928 |
| 11 | 0.151 | 0.257 | 0.295 | 0.209 | 0.172 | 0.060 | 0.370 | 0.330 | 0.123 | 0.140 |  | 0.545 | 1.301 | 0.711 | 1.512 | 0.758 | 0.304 | 1.052 |
| 12 | 0.279 | 0.219 | 0.195 | 0.156 | 0.241 | 0.338 | 0.209 | 0.202 | 0.273 | 0.281 | 0.314 |  | 1.595 | 1.659 | 0.591 | 1.096 | 0.472 | 0.493 |
| 13 | 0.167 | 0.156 | 0.169 | 0.061 | 0.100 | 0.173 | 0.238 | 0.167 | 0.130 | 0.119 | 0.161 | 0.136 |  | 2.129 | 1.476 | 1.622 | 0.707 | 0.926 |
| 14 | 0.259 | 0.158 | 0.133 | 0.075 | 0.235 | 0.275 | 0.204 | 0.143 | 0.223 | 0.213 | 0.260 | 0.131 | 0.105 |  | 0.756 | 1.588 | 0.758 | 0.660 |
| 15 | 0.109 | 0.221 | 0.259 | 0.206 | 0.113 | 0.152 | 0.328 | 0.339 | 0.085 | 0.129 | 0.142 | 0.297 | 0.145 | 0.248 |  | 0.826 | 0.333 | 1.279 |
| 16 | 0.232 | 0.113 | 0.092 | 0.181 | 0.196 | 0.290 | 0.237 | 0.173 | 0.221 | 0.225 | 0.248 | 0.186 | 0.134 | 0.136 | 0.232 |  | 0.918 | 0.865 |
| 17 | 0.422 | 0.292 | 0.248 | 0.299 | 0.393 | 0.486 | 0.423 | 0.328 | 0.423 | 0.394 | 0.451 | 0.346 | 0.261 | 0.248 | 0.429 | 0.214 |  | 0.353 |
| 18 | 0.130 | 0.222 | 0.236 | 0.257 | 0.157 | 0.240 | 0.383 | 0.326 | 0.173 | 0.212 | 0.192 | 0.336 | 0.213 | 0.275 | 0.163 | 0.224 | 0.414 |  |

Table S5 Estimation of Nei's genetic distance (above) and genetic identity (below) within 18 *B. striata* populations.

| Population | 01 | 02 | 03 | 04 | 05 | 06 | 07 | 08 | 09 | 10 | 11 | 12 | 13 | 14 | 15 | 16 | 17 | 18 |
| --- | --- | --- | --- | --- | --- | --- | --- | --- | --- | --- | --- | --- | --- | --- | --- | --- | --- | --- |
| 01 |  | 1.782 | 1.466 | 0.813 | 0.267 | 0.420 | 1.706 | 1.502 | 0.410 | 0.407 | 0.403 | 1.269 | 0.673 | 1.394 | 0.267 | 1.366 | 1.840 | 0.353 |
| 02 | 0.168 |  | 0.713 | 1.315 | 1.400 | 1.881 | 1.321 | 0.915 | 1.304 | 1.450 | 1.737 | 1.366 | 1.073 | 0.958 | 1.402 | 0.742 | 1.131 | 1.223 |
| 03 | 0.231 | 0.490 |  | 0.973 | 1.291 | 2.037 | 0.872 | 0.894 | 1.184 | 1.345 | 1.764 | 0.781 | 0.976 | 0.559 | 1.457 | 0.445 | 0.619 | 1.022 |
| 04 | 0.443 | 0.268 | 0.378 |  | 0.711 | 0.634 | 1.366 | 1.050 | 0.603 | 0.530 | 0.691 | 0.605 | 0.222 | 0.271 | 0.741 | 1.069 | 0.955 | 1.119 |
| 05 | 0.765 | 0.247 | 0.275 | 0.491 |  | 0.545 | 1.820 | 1.330 | 0.420 | 0.428 | 0.584 | 1.001 | 0.322 | 1.293 | 0.334 | 1.099 | 1.627 | 0.476 |
| 06 | 0.657 | 0.152 | 0.130 | 0.531 | 0.580 |  | 2.177 | 1.534 | 0.302 | 0.248 | 0.121 | 1.608 | 0.552 | 1.255 | 0.376 | 1.837 | 2.292 | 0.711 |
| 07 | 0.182 | 0.267 | 0.418 | 0.255 | 0.162 | 0.113 |  | 0.330 | 1.018 | 1.225 | 2.191 | 0.734 | 1.266 | 0.824 | 1.589 | 1.260 | 1.585 | 2.393 |
| 08 | 0.223 | 0.401 | 0.409 | 0.350 | 0.264 | 0.216 | 0.719 |  | 1.184 | 0.928 | 1.769 | 0.870 | 0.841 | 0.595 | 1.967 | 0.888 | 1.318 | 1.770 |
| 09 | 0.664 | 0.271 | 0.306 | 0.547 | 0.657 | 0.740 | 0.361 | 0.306 |  | 0.167 | 0.317 | 1.227 | 0.482 | 0.984 | 0.251 | 1.211 | 1.604 | 0.538 |
| 10 | 0.666 | 0.235 | 0.260 | 0.588 | 0.652 | 0.780 | 0.294 | 0.395 | 0.847 |  | 0.366 | 1.194 | 0.400 | 0.895 | 0.341 | 1.225 | 1.400 | 0.684 |
| 11 | 0.668 | 0.176 | 0.171 | 0.501 | 0.558 | 0.886 | 0.112 | 0.170 | 0.728 | 0.693 |  | 1.594 | 0.590 | 1.270 | 0.407 | 1.417 | 1.976 | 0.525 |
| 12 | 0.281 | 0.255 | 0.458 | 0.546 | 0.367 | 0.200 | 0.480 | 0.419 | 0.293 | 0.303 | 0.203 |  | 0.569 | 0.522 | 1.399 | 0.993 | 1.077 | 2.102 |
| 13 | 0.510 | 0.342 | 0.377 | 0.801 | 0.724 | 0.576 | 0.282 | 0.431 | 0.618 | 0.670 | 0.554 | 0.566 |  | 0.501 | 0.518 | 0.837 | 0.874 | 1.064 |
| 14 | 0.248 | 0.384 | 0.572 | 0.763 | 0.274 | 0.285 | 0.439 | 0.552 | 0.374 | 0.409 | 0.281 | 0.593 | 0.606 |  | 1.175 | 0.763 | 0.755 | 1.456 |
| 15 | 0.766 | 0.246 | 0.233 | 0.477 | 0.716 | 0.687 | 0.204 | 0.140 | 0.778 | 0.711 | 0.666 | 0.247 | 0.596 | 0.309 |  | 1.366 | 1.740 | 0.532 |
| 16 | 0.255 | 0.476 | 0.641 | 0.343 | 0.333 | 0.159 | 0.284 | 0.412 | 0.298 | 0.294 | 0.242 | 0.371 | 0.433 | 0.466 | 0.255 |  | 0.569 | 1.117 |
| 17 | 0.159 | 0.323 | 0.539 | 0.385 | 0.197 | 0.101 | 0.205 | 0.268 | 0.201 | 0.247 | 0.139 | 0.341 | 0.417 | 0.470 | 0.176 | 0.566 |  | 1.426 |
| 18 | 0.703 | 0.294 | 0.360 | 0.326 | 0.621 | 0.491 | 0.091 | 0.170 | 0.584 | 0.505 | 0.591 | 0.122 | 0.345 | 0.233 | 0.587 | 0.327 | 0.240 |  |


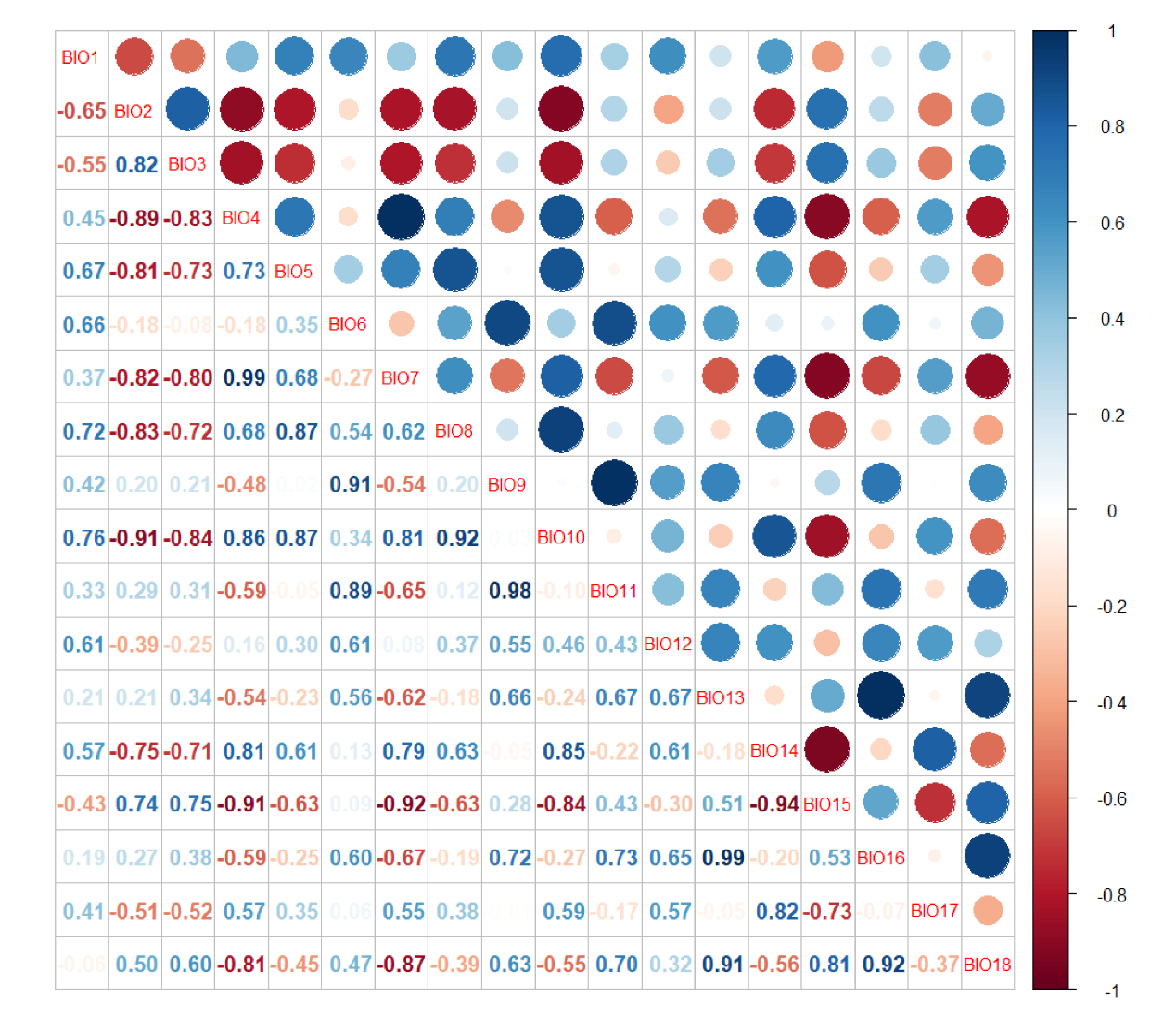


Figure S1. Analysis of the correlation among environmental variables. Blue stands for positive correlations while red represents negative correlations


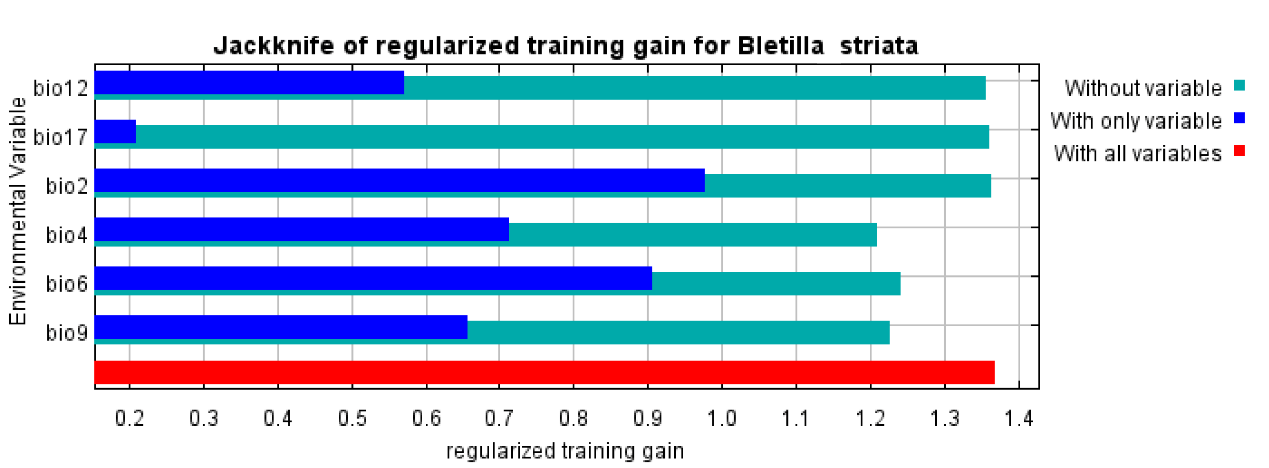


Figure S2. Results of Jackknife tests for the contribution of variables to the habitat distribution model of *B. striata*. The dark blue bars indicate the gain from using each variable in isolation, the light blue bars indicate the gain lost by removing the single variable from the full model, and the red bar indicates the gain using all of the variables.

**
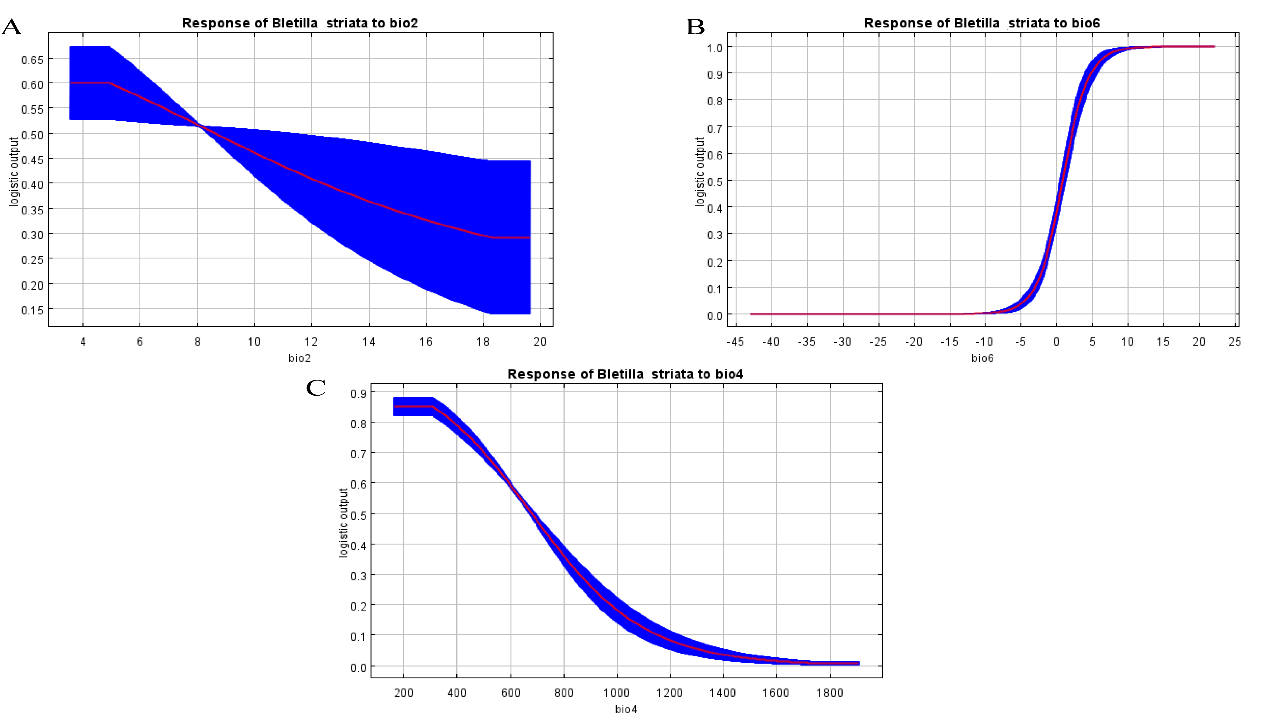
**

**Figure S3.** Response curves of the three main climatic variables to the existence probability of *B. striata*. The red line represents the mean value, and the blue area represents the range between the minimum and maximum values. A: BIO2 (mean diurnal range), B: BIO6 (min temperature of coldest month), C: BIO4 (temperature seasonality).

**
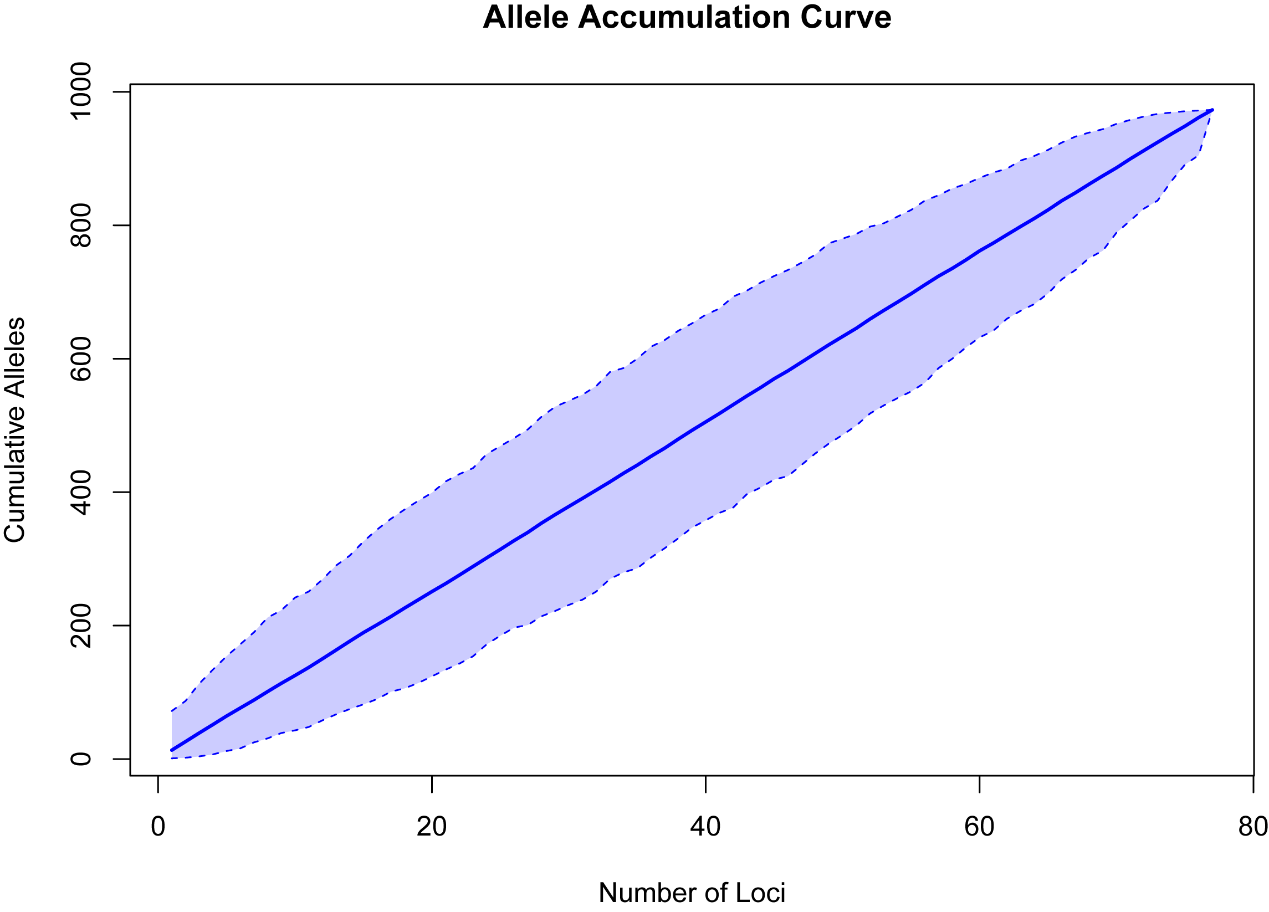
**

**Figure S4.** Rarefaction curve of allelic richness across eight SSR loci in *B. striata* populations.
